# Supplementary material for: Severe Radiation-Induced Lymphopenia Affects the Outcomes of Esophageal Cancer: A Comprehensive Systematic Review and Meta-Analysis
Source: Cancers (Basel). 2022 Jun 20;14(12):3024. doi: 10.3390/cancers14123024 (PMC9221375; doi:10.3390/cancers14123024)
Supplement: Supplementary file 1 [file cancers-14-03024-s001.zip › cancers-1735775-supplementary.pdf]

**Data S1.** Population, intervention, comparison, outcome and study design (PICOS) items and searching strategy in PubMed, Embase and Cochrane Library of current study.

**Table regarding to PICOS items**

| **Population** | **Patient with EC who had radiation therapy as a part of their therapy strategy** |
|---|---|
| Intervention | Patients with severe RIL who received radiation therapy for cancer |
| Comparison | Patients without severe RIL who received radiation therapy for cancer |
| Outcome | EC outcomes related data regarding to the association between severe RIL and non-severe RIL, such as pCR and survival related outcomes data |
| Study Design | Observational study (retrospective or prospective) |

EC, esophageal carcinoma; RIL, radiotherapy induced lymphopenia; pCR, pathologic complete response.

**Searching strategy in PubMed, Embase and Cochrane Library**

**Query #1**

(Lymphopenias OR Lymphocytopenia OR Lymphocytopenias OR Lymphopenia)

**Query #2**

(Radiotherapy OR radiation OR irradiat* OR x-ray-therap* OR chemoradiat* OR chemo-radiotherap* OR radiochemotherap* OR radio-chemotherap* OR Radiosurgery OR stereotactic OR SRT OR SRS OR SBRT OR SABR OR gamma knife OR GKRS OR LINAC OR CyberKnife OR Cyber Knife OR radioimmunotherap* OR immunoradiotherap* OR radio-immunotherap* OR immuno-radiotherap*)

**Query #3**

(Esophageal Neoplasm OR Neoplasm, Esophageal OR Esophagus Neoplasm OR Esophagus Neoplasms OR Neoplasm, Esophagus OR Neoplasms, Esophagus OR Neoplasms, Esophageal OR Cancer of Esophagus OR Cancer of the Esophagus OR Esophagus Cancer OR Cancer, Esophagus OR Cancers, Esophagus OR Esophagus Cancers OR Esophageal Cancer OR Cancer, Esophageal OR Cancers, Esophageal OR Esophageal Cancers)

**Final query = Query #1 AND Query #2 AND Query # 3**

We used the same query in PubMed, Embase and Cochrane Library.
The “All Fields” option was used in the search at PubMed.
The “Quick search” option was used in the search at Embase.
The “All text” option was used in the search at Cochrane Library.s

**Table S1.** NOS scale for cohort studies.

| Author/Year | Ref. | Selection | Comparabil-ity | Outcomes | NOS scores |
|---|---|---|---|---|---|
| Xu H/2021 | [16] | 4 | 1 | 3 | 8 |
| Wang Q/2021 | [28] | 4 | 1 | 3 | 8 |
| Nishida M/2021 | [29] | 4 | 1 | 3 | 8 |
| Liu M/2021 | [15] | 4 | 1 | 3 | 8 |
| Kroese T/2021 | [31] | 4 | 1 | 3 | 8 |
| Cai S/2021 | [30] | 4 | 0 | 3 | 7 |
| Xu C/2020 | [18] | 4 | 1 | 3 | 8 |
| Wang X/2020 | [17] | 4 | 1 | 3 | 8 |
| So T/2020 | [32] | 4 | 1 | 2 | 7 |
| Zhou X/2019 | [22] | 4 | 1 | 3 | 8 |
| Zhang E/2019 | [23] | 4 | 1 | 3 | 8 |
| Sherry A/2019 | [33] | 4 | 1 | 3 | 8 |
| Li Q/2019 | [19] | 4 | 1 | 3 | 8 |
| Deng W/2019 | [20] | 4 | 1 | 3 | 8 |
| Fang P/2018 | [34] | 4 | 1 | 3 | 8 |
| Routman D/2017 | [27] | 4 | 1 | 3 | 8 |
| Davuluri R/2017 | [21] | 4 | 1 | 3 | 8 |

Abbreviations: NOS, Newcastle-Ottawa Quality Assessment Form for Cohort Studies.

**Table S2.** Median ALC tendency for EC patients who received CRT.

| Study | Ref. | Pre-CRT ALC[a] | ALC nadir | Nadir time[b] | After CRT (4-8w) [ab] |
|---|---|---|---|---|---|
| Xu H/2021 | [16] | 1,800 | 300 | 4w | 1,200 |
| Nishida M/2021 | [29] | 1,469 | 354 | 6w | NA |
| Cai S/2021 | [30] | 1,400 | 300 | Post CRT | NA |
| Wang X/2020 | [17] | 1,730 | 450 | 5w | 970 |
| Zhou X/2019 | [22] | 1,425 | 340 | 6w | 715 |
| Li Q/2019 | [19] | 1,800 | 460 | 4w | 1,100 |
| Deng W/2019 | [20] | 1,480 | 280 | 5w | 860 |
| Fang P/2018 | [34] | 1,540 | 320 | 5w | NA |
| Davuluri R/2017 | [21] | 1,530 | 330 | 5w | NA |

Abbreviations: CRT, chemoradiation therapy; ALC, absolute lymphocyte count; NA, not available;.

[a]The unit of ALC was cells/μl;.

[b]The "w" represent "weeks".

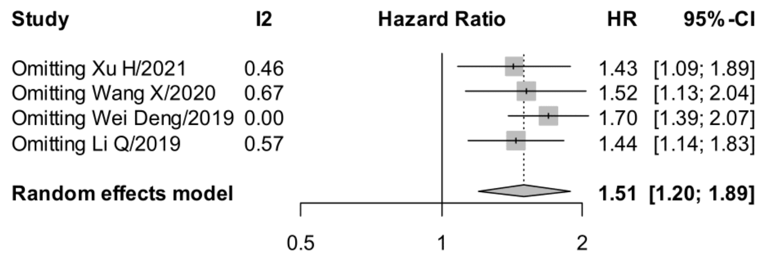


**Figure S1.** Forest plots of sensitivity analysis for meta-analysis on the association between severe RIL and the PFS of EC patients; sensitivity analysis for meta-analysis of the severe RIL and PFS of EC patients. For each line at the body of the plot, the HR and 95% CI correspond to the new pooled results after omitting a single study. The large diamond at the bottom of the plot represents the pooled HR of all studies. The width of the diamond represents 95% CI.
